# Reply to van Lingen et al. Comment on “Szczubełek et al. Effectiveness of Crohn’s Disease Exclusion Diet for Induction of Remission in Crohn’s Disease Adult Patients. *Nutrients* 2021, *13*, 4112”

**DOI:** 10.3390/nu14091734

**Published:** 2022-04-22

**Authors:** Martyna Szczubełek, Karolina Pomorska, Monika Korólczyk-Kowalczyk, Konrad Lewandowski, Magdalena Kaniewska, Grażyna Rydzewska

**Affiliations:** 1 Clinical Department of Internal Medicine and Gastroenterology with Inflammatory Bowel Disease Subunit, Central Clinical Hospital of Ministry of the Interior and Administration, 02-507 Warsaw, Poland; k.m.pomorska@gmail.com (K.P.); monika.korolczyk@o2.pl (M.K.-K.); dr.k.lewandowski@icloud.com (K.L.); gastroenterologia@cskmswia.gov.pl (M.K.); grazyna.rydzewska@cskmswia.gov.pl (G.R.); 2 Collegium Medicum, Jan Kochanowski University, 25-317 Kielce, Poland

Thank you for your comment and interest [1] in our manuscript [2]. I would like to discuss the differences obtained in our research results. 

Our study was met with great interest among IBD patients, which translated into good compliance during the study. Perhaps this was related to the different availability of medical care in our countries. During the COVID-19 pandemic, access to specialised medical care in Poland was significantly limited. When patients were included in the study, they received regular in-person appointments with a full medical examination, laboratory tests and a dietician consultation. In Poland, access to high-class clinical nutritionists is significantly difficult. In addition, we provided Modulen formula free of charge, the regular cost of which exceeded the capabilities of many patients. Considering the benefits, the widespread fear of the COVID-19 pandemic, while at the same time ensuring constant access to medical care, it seems that our patients were not only willing to participate in the study but were also willing to follow the recommendations.

Regarding the concentration of calprotectin—we observed a statistically significant decrease in calprotectin level after 12 weeks of dietary intervention. The differences between week 0 vs. week 6 were not significant. However, clinical improvement can usually be observed before mucosal healing and this is consistent with our study results.

The effectiveness of Crohn’s disease exclusion diet (CDED) seems to encourage its use in CD patients. We also see great value in conducting a randomized, controlled trial that would provide high-value evidence for the efficacy of CDED. We will be very happy to conduct such a study in the future.

Thank you once again for your comment and discussion.

## Data Availability

Data supporting reported results are available upon request.
